# Concurrent amebic and cytomegalovirus colitis in immunocompromised patients: two case reports and literature review

**DOI:** 10.3389/fmed.2026.1751023

**Published:** 2026-04-29

**Authors:** Chen-Hsuan Lin, Li-Teh Liu, Yi-Hsuan Chen, Peir-In Liang, Yao-Kuang Wang, Jih-Jin Tsai

**Affiliations:** 1 School of Medicine, College of Medicine, Kaohsiung Medical University, Kaohsiung City, Taiwan; 2 Department of Medical Laboratory Science and Biotechnology, College of Medical Technology, Chung Hwa University of Medical Technology, Tainan City, Taiwan; 3 Department of Medical Education, Kaohsiung Medical University Hospital, Kaohsiung City, Taiwan; 4 Department of Pathology, Kaohsiung Medical University Hospital, Kaohsiung City, Taiwan; 5 Division of Gastroenterology, Department of Internal Medicine, Kaohsiung Medical University Hospital, Kaohsiung City, Taiwan; 6 Tropical Medicine Center, Kaohsiung Medical University Hospital, Kaohsiung City, Taiwan; 7 Division of Infectious Diseases, Department of Internal Medicine, Kaohsiung Medical University Hospital, Kaohsiung City, Taiwan

**Keywords:** bloody diarrhea, case report, cytomegalovirus colitis, *Entamoeba histolytica*, immunocompromised host, mixed infection

## Abstract

Immunosuppression from human immunodeficiency virus (HIV) or other comorbidities increases susceptibility to gastrointestinal infections such as *Entamoeba histolytica* and cytomegalovirus (CMV). Although each pathogen can independently cause severe colitis, co-infection is rare and may lead to rapid clinical deterioration if not recognized early. We report two cases of concurrent amebic and CMV colitis in immunocompromised patients. The first patient, a 56-year-old man with HIV, Kaposi sarcoma, and hepatitis C, presented with bloody diarrhea and hypotension. Colonoscopy revealed pancolitis with ulcerations, and biopsy initially confirmed *E. histolytica*. Despite an initial response to anti-amebic therapy, he later developed recurrent bleeding and sepsis and died on hospital day 24. Postmortem pathological examination subsequently identified CMV co-infection. The second patient, a 48-year-old HIV-negative man with diabetes, chronic hepatitis B and C, and prior colorectal cancer, presented with 1 month of bloody diarrhea. Colonoscopy and histopathology confirmed dual infection, which was successfully treated with metronidazole and ganciclovir. A follow-up colonoscopy later demonstrated complete resolution. These cases highlight the importance of early endoscopic evaluation and tissue diagnosis in immunocompromised patients with persistent bloody diarrhea, as prompt recognition of co-infection allows timely, targeted therapy and may prevent fatal outcomes.

## Introduction

Human immunodeficiency virus (HIV) progressively impairs cellular immunity through depletion of CD4-positive T cells, placing affected individuals at increased risk for opportunistic infections. The gastrointestinal tract is one of the earliest and most frequently affected organ systems in HIV infection, and infectious colitis remains a major cause of morbidity in immunocompromised patients (1). Among the various pathogens implicated, *Entamoeba histolytica* and cytomegalovirus (CMV) are two clinically important organisms capable of causing severe colonic inflammation (2).

Infection with *E. histolytica* ranges from asymptomatic colonization to invasive amebic colitis, which may present with abdominal pain, diarrhea, and, in severe cases, fulminant hemorrhagic colitis or intestinal perforation (3). Although traditionally associated with fecal-oral transmission in endemic regions, invasive amebiasis has increasingly been recognized in non-endemic settings, particularly among vulnerable or immunocompromised populations (4). Multiple mucosal ulcerations may occur, predisposing patients to extensive bleeding and systemic complications.

CMV colitis predominantly affects individuals with impaired cellular immunity, particularly those with advanced HIV infection (5). CMV infection can cause deep mucosal ulceration, edema, and tissue necrosis, potentially resulting in massive gastrointestinal bleeding, intestinal perforation (6), or obstructive inflammatory masses (7). CMV-induced mucosal injury may also facilitate secondary infections, including bacterial translocation or superimposed pathogens such as *E. histolytica*, thereby exacerbating disease severity (8, 9).

Concurrent infection with *E. histolytica* and CMV involving the colon has rarely been reported, particularly outside endemic regions. Most available evidence consists of isolated case reports describing dual infection in immunocompromised patients rather than large epidemiological studies (2, 5, 6, 9–14), suggesting that the global incidence of this co-infection is low (Table 1). Although infectious colitis is common in advanced HIV infection, co-infection involving multiple opportunistic pathogens appears to be uncommon and may be underrecognized in clinical practice (15).

**Table 1:** Reported cases of concurrent *E. histolytica* and CMV colitis in the literature, including the two cases from the present study.

Study	Country	Patient	Immunocompromise	Key findings	Outcome
Fätkenheuer et al. (1997) (10)	Germany	33, 40 M	AIDS	Amebic + CMV colitis	2 survived
Hung et al. (1999) (13)	Taiwan	27, 32, 35, 44 M	AIDS (CD4 10–147/μL, naïve, without ART)	Amebic + CMV colitis	2 survived, 2 death
Lee et al. (2004) (5)	USA	29 M	None (immunocompetent)	Amebic + CMV colitis	Survived
Tsai et al. (2005) (6)	Taiwan	34 M	AIDS (CD4 18/μL, VL 2,641 copies/mL, naïve, without ART)	Amebic + CMV colitis, cecal perforation	Survived
Matsumoto et al. (2011) (14)	Japan	38, 44, 46, 52, 58, 62 M	AIDS (CD4 5–422/μL), severe malnutrition	Amebic + CMV colitis, 2 perforations (cecal, ascending)	4 survived, 2 death
Weng et al. (2016) (12)	Taiwan	50 M	AIDS (CD4 0, VL 141,000 copies/mL, naïve, without ART), steroids	Amebic + CMV colitis, multiple perforations	Death
Diaz et al. (2018) (11)	USA	70s M	Heavy metal exposure	Amebic + CMV colitis, perforation	Survived
Morikubo et al. (2018) (2)	Japan	40 M	AIDS (CD4 138.9/μL, naïve, without ART)	Amebic + CMV colitis	Survived
Shijubou et al. (2021) (9)	Japan	68 M	Steroids, T2DM	Fulminant amebic + CMV colitis, perforation	Death
This study	Taiwan	56 M	AIDS (CD4 41.6/μL, VL 3,050 copies/mL, recent non-adherence to ART), IVDU	Hemorrhagic amebic + CMV colitis	Death
		48 M	T2DM, chronic alcohol use, prior colon adenocarcinoma	Amebic + CMV colitis	Survived

AIDS, acquired immunodeficiency syndrome; CMV, cytomegalovirus; ART, antiretroviral therapy; VL, viral load; T2DM, type 2 diabetes mellitus; IVDU, intravenous drug use.

Despite characteristic clinical and endoscopic features, the manifestations of amebic and CMV colitis often overlap, typically presenting with persistent bloody diarrhea, fever, abdominal pain, and weight loss (16, 17). Because treatment strategies differ—anti-parasitic therapy for amebiasis and antiviral therapy for CMV—failure to identify mixed infection may delay appropriate management and worsen clinical outcomes (9).

We present two cases of concurrent amebic and CMV colitis in immunocompromised patients. The first case describes an HIV-infected patient who developed severe hemorrhagic colitis and ultimately died of hypovolemic shock despite treatment. The second case illustrates successful recovery following early recognition and pathogen-specific therapy. These cases emphasize the importance of timely evaluation and consideration of dual infection in immunocompromised patients presenting with severe or persistent bloody diarrhea.

## Case description

### Patient 1

A 56-year-old man with a history of acquired immunodeficiency syndrome (AIDS), Kaposi sarcoma, chronic hepatitis C, and intravenous drug use (IVDU) was transferred to the emergency department after sudden loss of consciousness. On arrival, he was hypotensive and required endotracheal intubation and vasopressor support. Coffee-ground nasogastric aspirate suggested upper gastrointestinal bleeding. Bilateral lower-extremity ecchymoses were found on physical examination.

Laboratory evaluation showed anemia, thrombocytopenia, lymphopenia, coagulopathy, acute kidney injury, and metabolic acidosis. The patient had been receiving antiretroviral therapy (ART) consisting of dolutegravir, abacavir, and lamivudine. He had maintained good control on ART for the past 10 years, with a CD4 count of 449 cells/μL and an HIV viral load of 44.9 copies/mL 3 months prior. On admission, his CD4 count had declined to 41.6 cells/μL and viral load increased to 3,050 copies/mL, suggesting recent non-adherence, possibly related to IVDU.

Computed tomographic angiography revealed a hypervascular hepatic lesion, prompting transcatheter arterial embolization for suspected bleeding. The patient was subsequently admitted to the intensive care unit and treated empirically with broad-spectrum antibiotics.

**Figure 1:**
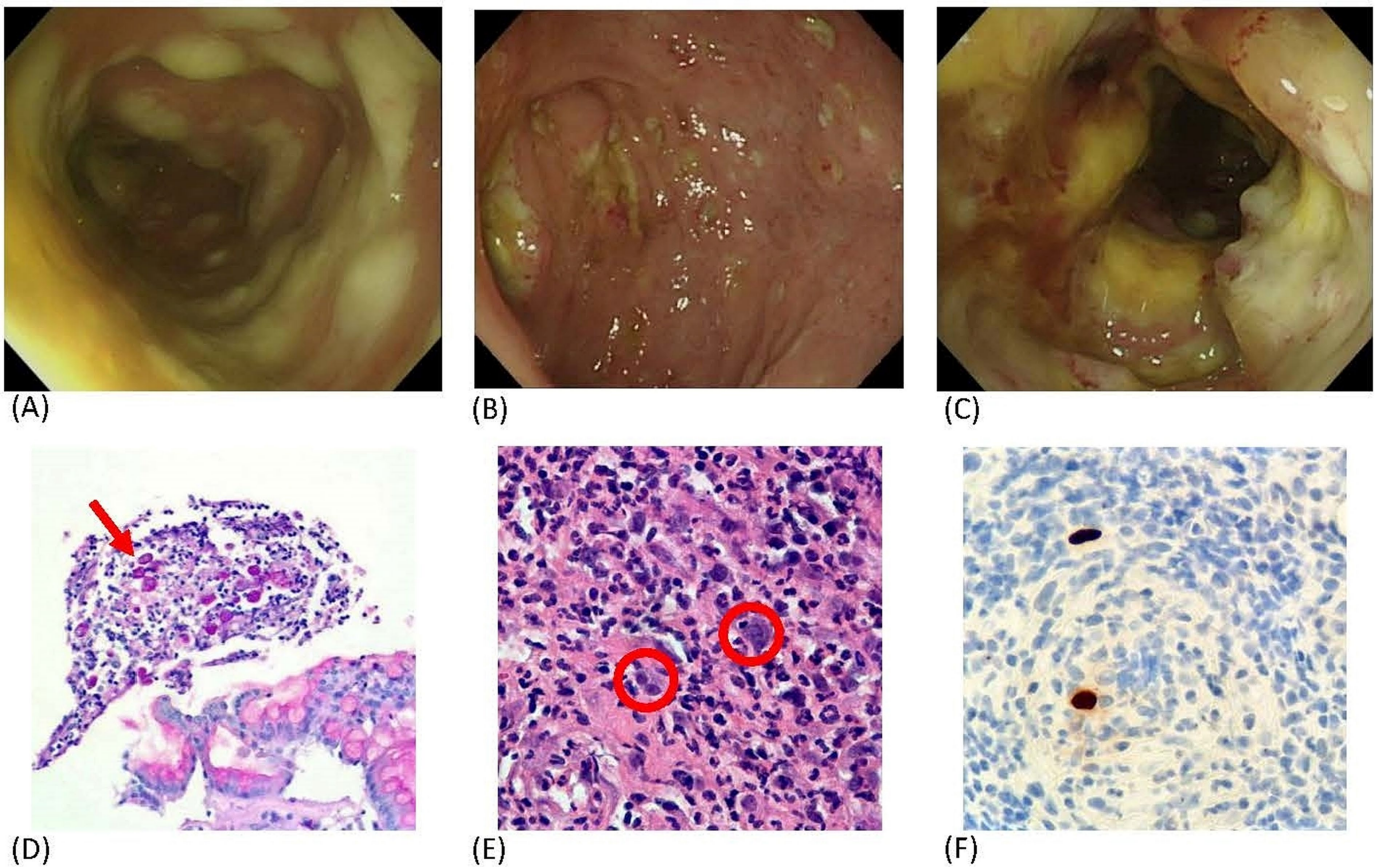
Colonoscopic and histopathological findings. Colonoscopic images show **(A)** diffuse elevated white plaques on the mucosal surface of the descending and sigmoid colon, **(B)** multiple punched-out ulcers extending from the cecum to the sigmoid colon, and **(C)** a circumferential nodular lesion with an ulcerated surface in the sigmoid colon. Histopathology demonstrates **(D)** *E. histolytica* trophozoites containing ingested erythrocytes in the mucosa (PAS stain), **(E)** giant cells with characteristic intranuclear “owl’s eye” inclusions, and **(F)** positive immunohistochemical staining for cytomegalovirus.

On post-symptom onset (PSO) day 7, he developed bloody diarrhea and fever. Colonoscopy on PSO day 10 revealed pancolitis with multiple ulcerations extending from the cecum to the rectum (Figures 1A–C). Biopsy identified *E. histolytica*, and metronidazole was initiated. Stool examinations for ova and parasites were repeatedly negative. Polymerase chain reaction (PCR) and serology by indirect hemagglutination assay (IHA) were both positive for *E. histolytica*. Later, paromomycin was added for luminal eradication. CMV polymerase chain reaction (PCR) testing showed a viral load of 1,630 IU/mL. Because CMV was not confirmed histologically at that time and the viral load was relatively low, antiviral therapy was not initiated.

Despite an initial response to anti-amebic therapy, he later developed recurrent fever and repeated episodes of bloody diarrhea. On PSO day 24, the patient developed massive gastrointestinal bleeding leading to hypovolemic shock and cardiac arrest, and resuscitation was unsuccessful. Postmortem review of the colonoscopic biopsy with additional immunohistochemical staining revealed both PAS-positive *E. histolytica* trophozoites containing ingested erythrocytes and characteristic CMV intranuclear “owl’s eye” inclusions with positive CMV immunostaining (Figures 1D–F), confirming concurrent amebic and CMV colitis. Extensive mucosal destruction caused by both pathogens was considered the primary cause of fatal hemorrhagic colitis.

### Patient 2

A 48-year-old man presented with a one-month history of bloody diarrhea without fever or abdominal pain. His medical history included type 2 diabetes mellitus, chronic alcohol use, chronic hepatitis B and C, and previously resected sigmoid colon adenocarcinoma. HIV testing was negative.

Colonoscopy revealed diffuse colitis with mucosal ulcerations. Histopathological analysis of colonoscopic biopsy showed PAS-positive protozoan trophozoites containing ingested erythrocytes, consistent with *E. histolytica*. In addition, atypical cells with eosinophilic intranuclear inclusion bodies were observed and confirmed by CMV immunohistochemical staining, establishing concurrent CMV infection. Stool examination did not identify amebic cysts or trophozoites. Given definitive histopathological findings, PCR for *E. histolytica* was not performed; however, serology by IHA was positive.

Intravenous metronidazole and ganciclovir were initiated. The patient’s diarrhea improved rapidly with treatment. Ganciclovir was transitioned to oral valganciclovir on hospital day 3, and he was discharged in stable condition. Paromomycin was prescribed at follow-up to complete luminal eradication of amebiasis. One month later, repeat colonoscopy showed complete resolution of ulceration and mucosal inflammation, with no residual colitis or stenosis.

## Discussion

HIV-associated immunosuppression predisposes patients to a wide spectrum of gastrointestinal infections, particularly as CD4 counts decline. CMV is a well-recognized opportunistic pathogen in advanced HIV infection and typically emerges when CD4 counts fall below 50 cells/mm^3^ (18). Although CMV retinitis is the most common manifestation of CMV end-organ disease, CMV colitis represents an important gastrointestinal complication (19). Patients commonly present with abdominal pain, diarrhea, hematochezia, and weight loss (20). Endoscopically, CMV colitis may appear as deep punched-out ulcers or diffuse mucosal inflammation, often resembling other infectious colitides, which makes histopathological confirmation essential for diagnosis. CMV should therefore be considered in the differential diagnosis of unexplained bloody diarrhea in patients with advanced HIV infection.

Amebic colitis caused by *E. histolytica* is also observed more frequently among individuals with HIV infection (14, 20). Although it is not classified as an opportunistic pathogen, disease severity may be increased in immunocompromised hosts (21). Severe cases may present with extensive ulceration, hematochezia, and, in rare instances, toxic megacolon (22). Because clinical and endoscopic findings overlap with those of other colitides, diagnostic confirmation typically relies on stool antigen testing, serological assays, or histopathological examination.

Patients without HIV may also develop clinically significant immunocompromise due to metabolic and lifestyle-related conditions. Type 2 diabetes mellitus has been associated with weakened innate and adaptive immune responses, including reduced chemotaxis, impaired phagocytosis, dysregulated cytokine production, and altered T-cell immunity (23). Chronic alcohol use can similarly blunt host defense by disrupting gut microbiota and increasing intestinal permeability through epithelial tight junction damage, promoting mucosal inflammation and susceptibility to gastrointestinal infections and colitis (24). IVDU may further contribute via increased pathogen exposure and drug-induced immunomodulation (25). Cancer patients often exhibit systemic immune defects, including cytopenias, T- and B-cell suppression, and expansion of immunosuppressive cell populations, contributing to impaired immune function (26).

Several case reports have described severe outcomes associated with *E. histolytica* and CMV co-infection, including colonic perforation (6), massive hemorrhage, or intestinal stenosis requiring surgery (9). Proposed mechanisms include CMV-induced mucosal injury facilitating secondary invasion by *E. histolytica*, or conversely, pre-existing amebic ulceration promoting CMV reactivation within damaged mucosa (5).

The coexistence of amebic and CMV colitis is generally uncommon, with most reports limited to isolated cases. A retrospective study from Japan, a non-endemic region, found that 31.6% (6/19) of patients with amebic colitis had concurrent CMV enteritis; however, because it was a single-center study, these findings did not reflect the global epidemiology (14). Notably, co-infection was markedly more frequent in HIV-positive patients (71.4%, 5/7) compared with HIV-negative patients (11.1%, 1/9), suggesting that immunodeficiency, rather than geographic exposure, plays a key role in its development. Nevertheless, comprehensive global data remain limited.

Consistent with this, a review of the literature over the past three decades identified only 20 reported cases of concurrent amebic and CMV colitis (including the present 2 cases), as summarized in Table 1. Among these, 16 occurred in patients with AIDS, with a mortality rate of 37.5% (6/16), compared with 33.3% (1/3) in non-AIDS immunocompromised patients and no deaths in the single immunocompetent case. These findings further highlight both the rarity of co-infection and its substantial mortality, particularly among patients with advanced HIV infection.

In our first patient, CMV infection was recognized only after postmortem re-evaluation of the colonoscopic biopsy. Because the initial pathology identified only *E. histolytica*, and CMV viremia was low, CMV colitis was not strongly suspected, and antiviral therapy was thus not initiated. Earlier recognition of CMV disease might have allowed timely antiviral treatment and could have altered the clinical course. However, systemic CMV viremia is an unreliable indicator of gastrointestinal end-organ disease due to its low sensitivity; therefore, diagnosis and initiation of antiviral therapy generally rely on histopathological confirmation of tissue-invasive disease (27). Nevertheless, a recent case report described clinical improvement after early initiation of ganciclovir in a patient with AIDS when CMV colitis was strongly suspected based on a positive serum PCR result (28). In severely immunocompromised patients with persistent or worsening gastrointestinal bleeding despite appropriate antimicrobial therapy, repeat endoscopic evaluation or reassessment of biopsy specimens may help exclude concurrent CMV infection and facilitate earlier targeted treatment.

## Conclusion

Concurrent *E. histolytica* and CMV colitis is rare but may lead to severe gastrointestinal complications in immunocompromised patients. Because the clinical and endoscopic features of these infections often overlap, dual infection may be overlooked without careful diagnostic evaluation. Early colonoscopic assessment with histopathological confirmation is therefore essential in patients presenting with persistent or unexplained bloody diarrhea.

Timely identification of both pathogens allows prompt initiation of targeted antimicrobial and antiviral therapy, which may significantly improve clinical outcomes. Clinicians should maintain a high index of suspicion for co-infection in severely immunocompromised patients, particularly those with advanced HIV infection or refractory colitis despite appropriate treatment.

## Data Availability

The raw data supporting the conclusions of this article will be made available by the authors, without undue reservation.
